# Experimental Validation of Antiobesogenic and Osteoprotective Efficacy of Ginsenoside CK via Targeting Lipid and Atherosclerosis Pathways

**DOI:** 10.3390/life15010041

**Published:** 2024-12-31

**Authors:** Md. Niaj Morshed, Reshmi Akter, Imran Mahmud, Ah-Yeong Gwon, Jin Woo Jeang, Yeong-Geun Lee, Dae Won Park, Deok Chun Yang, Yeon Ju Kim, Se-Chan Kang

**Affiliations:** 1 Department of Biopharmaceutical Biotechnology, College of Life Science, Kyung Hee University, Yongin-si 17104, Gyeonggi-do, Republic of Korea; niajmorshed96@khu.ac.kr; 2 Graduate School of Biotechnology, College of Life Sciences, Kyung Hee University, Yongin-si 17104, Gyeonggi-do, Republic of Korea; reshmiakterbph57@gmail.com (R.A.); kbg02076@naver.com (A.-Y.G.); jinjang1218@nmr.kr (J.W.J.); lyg629@khu.ac.kr (Y.-G.L.); parkdw@genencell.co.kr (D.W.P.); dcyang@khu.ac.kr (D.C.Y.); 3 Department of Biochemistry & Biotechnology, University of Turku, 20014 Turku, Finland; imrmah@utu.fi

**Keywords:** obesity, osteoporosis, ginsenoside CK, inflammation, lipid and atherosclerosis

## Abstract

The present study explored the possible antiobesogenic and osteoprotective properties of the gut metabolite ginsenoside CK to clarify its influence on lipid and atherosclerosis pathways, thereby validating previously published hypotheses. These hypotheses were validated by harvesting and cultivating 3T3-L1 and MC3T3-E1 in adipogenic and osteogenic media with varying concentrations of CK. We assessed the differentiation of adipocytes and osteoblasts in these cell lines by applying the most effective doses of CK that we initially selected. Using 3T3-L1 adipocytes in vitro assessments, CK could effectively decrease intracellular lipid accumulation, inhibit α-glucosidase enzyme, increase 2-NBDG glucose uptake, reduce inflammation-associated cytokines (*TNFα*, and *IL-6*), adipogenic regulatory genes (*PPARγ*, *FAS*, *C/EBPα*), lipogenic gene *LPL*, and increase the expression of thermogenic gene *UCP1*. Additionally, CK treatment induced osteoblast development in MC3T3-E1 cells as shown by increased mineralization and calcium distribution, collagen content, alkaline phosphatase activity, and decreased inflammatory cytokines *TNFα*, and *IL-6* and increased the regulated expressions of osteogenic genes including *Runx2*, *ALP*, *BGLAP*, *OCN*, and *Col1a1*. Significantly, as a major inhibitory regulator, the *TP*^53^ gene was down-regulated in both 3T3-L1 and MC3T3E1 cells after the treatment of CK. These encouraging results demonstrate the possible use of CK as an innovative treatment for controlling obesity and osteoporosis, targeting the underlying mechanisms of obesogenic and bone loss. Further studies are necessary to explore the clinical implications of these results and the potential of CK in future treatment strategies. This research highlights the promise of CK in addressing significant health issues.

## 1. Introduction

Obesity can be defined by high-fat production, which may negatively affect health. By 2030, the global obesity rate is expected to exceed 1 billion, with 15% of men and 20% of women being obese. Several diseases, including diabetes, osteoarthritis, cardiovascular conditions, and various forms of cancer, are related to obesity and thereby increase the death rate. An estimated 2.8 million deaths worldwide are attributed to obesity and being overweight [1]. The inception and progression of obesity depends on micro-environmental, genetic, metabolic, and behavioral variables [2]. The disparity between greater consumption of food and reduced expenditure of energy leads to changes in the distribution and release of fatty tissue [3]. Excessive fat storage leads to the hypertrophy of adipocytes, which in turn causes the adipose tissue to release more proinflammatory mediators, including *TNF-α*, *IL1β*, *IL6*, *resistin*, and *leptin*. Hypoxic conditions caused by a lack of blood vessels and M1-like macrophage infiltration promote adipose tissue inflammation [4,5,6]. Adipose tissue-derived mediators are released into the bloodstream, leading to a low-grade inflammatory condition throughout the body, which spreads to other organs, such as the liver, pancreas, hypothalamus, bone, and skeletal muscle [7]. Obesity can impair the bone remodeling balance, deregulate bone homeostasis, and promote bone loss because of the systemic inflammatory state. Two mechanisms primarily cause this condition: (i) obese adipose tissue releases proinflammatory mediators, which increase osteoclast development and resorption while decreasing osteoblast activity; and (ii) obesity-induced reduction in MSC differentiation toward the osteogenic lineage, while enhancing adipogenic differentiation [8].

For more than two centuries, Panax ginseng Meyer (Korean ginseng) has been regarded as one of the most successful traditional Chinese herbal remedies. Its pharmacological qualities are mostly ascribed to its potent triterpenoid saponins. Numerous parts of the ginseng plant, including the roots, flowers, berries, and leaves, have yielded more than 218 ginsenosides, and these byproducts are now widely used in research. However, ginsenoside component K (CK) is one of the most essential among these ginsenosides because of its high uptake and rate of absorption through the gastrointestinal tract of humans and, eventually, the systemic circulation. CK is a low-natural-abundance intestinal microbial metabolite mostly generated in the gut through metabolic transformation, side chain modification, or physicochemical processing. Furthermore, CK has stronger biological activity than main ginsenosides, which has caused issues with ginseng advancement as well as nutritional supplements and natural foods containing ginsenosides. Several bio-conversion mechanisms utilize ginsenosides Rb1, Rb2, and Rc as a substrate to produce CK. CK has many pharmacological effects, such as increasing bone development, lipid and glucose metabolism, oxidation of lipids, insulin resistance, anti-inflammatory properties, and anti-apoptotic qualities. Its therapeutic use can be advanced by more investigation into the toxicity and bioavailability of CK [9]. After performing clinical trials, researchers confirmed that CK is effective and well-tolerated in single doses that varied from 100 to 400 mg, and showed a gradual increase in Cmax and AUC levels in young Chinese volunteers [10]. Moreover, it has been reported that CK inhibits obesity and osteoporosis by regulating several signaling pathways. CK treatment significantly reduces the lipid droplets and adipogenesis through the down regulations of *PPARγ*, *SREBP1*, *adiponectin*, *FABP4*, and *C/EBPα* mRNA expression and *PPARγ*, *FABP4*, and *C/EBPα* protein levels via AMPK, AKT, and MAPK signaling pathways [11]. Another study showed that CK substantially suppressed adipogenesis, which could be mediated in part by alterations in the functioning of genes involved in angiogenesis (*VEGF-A* and *FGF-2*) and the MMPs (*MMP-2*, and *MMP-9*). Thus, by inhibiting adipogenesis, compound K may have beneficial effects for the prevention and management of obesity and related conditions [12]. On the other hand, Ding et al. found that CK activates the Wnt/β-catenin signaling pathway, promoting osteogenesis using immunofluorescence labeling and luciferase reporter assays [13]. Furthermore, CK significantly reduced the expression of genes linked to inflammation, such as *IKK* and interleukin 1β (*IL-1β*), while increasing the expression of osteogenic markers, such as *ALP* and *Col-I* in MC3T3-E1 cells stimulated with H_2_O_2_ [14]. However, many more plausible pathways remain unknown. We attempted to identify a novel signaling mechanism that is effective for both obesity and Osteoporosis.

Previously, we have reported new insights into microbial metabolites-based medications or dietary supplementations to prevent obesity and osteoporosis through network pharmacology and several computational techniques [15]. In that study, the lipid and atherosclerosis were identified as a key Kyoto Encyclopedia of Genes and Genomes (KEGG) pathway involved in the protective effects of microbial metabolite ginsenoside Compound K (CK) on obesity and osteoporosis. Moreover, we analyzed the molecular docking data and found that CK and TP^53^ showed a higher negative binding affinity than other bonds for both diseases. According to molecular dynamic simulation, the CK showed strongly stable RMSD and RMSF values. Additionally, CK has higher ROG and SASA values against TP^53^. Therefore, the current research performed an in vitro study to further examine whether CK counteracts the effect of TP^53^ on obesity and osteoblast.

## 2. Materials and Methods

### 2.1. Reagents and Chemicals

We received ginsenoside CK (biotransformed by *Lactobacillus paralimentarius*, purity 88% [16]) from the Hanbang Bio Lab in South Korea. 3T3-L1-murine pre-adipocyte and MC3T3-E1 pre-osteoblast cell lines were obtained from ATCC (American Type Culture Collection). Welgene, South Korea, supplied the following: FBS (fetal bovine serum), BCS (bovine calf serum), α-MEM (Alpha Modification of Eagle’s Minimum Essential Media), and DMEM (Dulbecco’s Modified Eagle’s Medium). Dexamethasone, IBMX (3-isobutyl-1-methylxanthine), and penicillin–streptomycin were acquired from Wako, Japan, and GeneDEPOT, respectively. The sources of acarbose, insulin, rosiglitazone, and 17β-estradiol (E2) were Sigma Aldrich Chemicals, St. Louis, MO, USA. α-glucosidase, P-NPG and 2-NBDG were acquired from Thermo Fisher Scientific (Waltham, MA, USA).

### 2.2. Cell Culture

A complete medium (CM) comprising 10% BCS and 1% P/S in DMEM was used to seed 3T3-L1 fibroblast pre-adipocytes. Furthermore, MC3T3-E1 pre-osteoblast were seeded in media containing 10% FBS and 1% P/S with α-MEM. The cells were then incubated with 5% CO_2_ at 37 °C.

### 2.3. Cell Viability Assay

An optical measurement assay based on MTT absorption by live cells was used to evaluate growth in 3T3L1 and MC3T3-E1 cells, respectively. Cells were cultured at 1 × 10^4^ cells per well in 96-well plates. Following a two-hour incubation, samples of varied quantities were introduced to the cells and incubated for another 24 h. After the incubation, 20 µL of MTT solution was added to each well and the plates were permitted to incubate for another three to four hours. Finally, the absorbance was evaluated at 570 nm after applying 100 μL of DMSO. We calculated the relative cell vitality (%) by comparing the absorbance of the treated cells to that of the control cells (untreated).

### 2.4. Differentiation of Adipocytes in 3T3-L1 Cells

The 3T3-L1 pre-adipocyte cell line was seeded in a 75 cm^2^ culture flask with CM and incubated until approximately 80% confluence was reached. Differentiation was then induced following the protocol outlined by Morshed et al. [17]. Subsequently, the cells were plated in a 12-well at a density of 1 × 10^4^ cells/well plate with a basal medium.

The cells were incubated for 48 h until 70% viability was achieved (Day 0). At this point, the medium was replaced with a basal medium supplemented with Differentiation Medium I (MDI), which contained dexamethasone (1 µM), insulin (10 µg/mL), and IBMX (0.5 mM). The cells were then cultured for another 48 h. After this incubation period, the medium was refreshed on Days 2 and 4 with a basal medium containing insulin. On Day 6, the medium was changed again to basal medium, which was further supplemented with CK and Rosiglitazone (RSG) at various concentrations.

### 2.5. Lipid Formation and Triglyceride Determination Assay

After differentiation, an Oil Red O assay was conducted to visualize lipid droplets, which are indicative of lipid droplets in 3T3-L1 cells. The cells were first rinsed with 1× PBS (sodium buffered saline) and then fixed in formalin (10%) for several hours or days. After fixation, the cells were stained with ORO working solution for 15 min, and the excess stain was removed by rinsing with distilled water. Phenotypic changes in the fully differentiated adipocytes were observed using an inverted light microscope (Nikon Instruments, Melville, NJ, USA). To assess triglyceride content and fat storage capacity, 100% isopropanol was utilized in the adipocytes, and the absorbance at 520 nm was measured after incubating the cells for 10 min at room temperature [18].

### 2.6. Inhibitory Effects of α-Glucosidase

A slight modification of the previously published method [17] was used to assess the inhibitory activity of α-glucosidase. To put it briefly, α-GLU solid powder was added to 0.1 M PBS to make 1 U/mL of a-GLU working solution. The reaction was started by adding 50 mL of CK at different concentrations (2.5, 5, and 10 μM) or Acarbose as standard at the same concentration and 100 mL of α-GLU working solution on a 96-well plate, which was then incubated at 37 °C for 10 min. 100 mL of a 5 mM p-NPG solution was then added, and the mixture was incubated for 20 min at 37 °C. After the reaction was ultimately halted by adding 200 mL of 0.5 M sodium carbonate solution, the absorbance value was determined at 405 nm. The formula [{(Control abs-Sample abs)/Control abs} × 100] was used to obtain the inhibition rate (%). The IC_50_ values were also calculated by using a standard curve.

### 2.7. 2-NBDG Uptake in 3T3L1 Cells

A dye-based glucose derivative, 2-NBDG, has been utilized to determine the level of glucose uptake following some modifications to the previously reported technique [17]. 3T3-L1 adipocytes were seeded in 96-well plates using a glucose-free medium containing 10% FBS. After a 24 h incubation period, cells were treated with or without 50 mM 2NBDG using CK in various doses (2.5, 5, and 10 μM), and insulin (100 nM) as a positive control.

### 2.8. Osteoblast Differentiation and Alkaline Phosphatase (ALP) Activity

Osteoblast differentiation and the ALP assay were conducted using a previously published study [19]. In brief, MC3T3-E1, pre-osteoblast cells were cultured into 12-well plates and were allowed to reach an 80 to 90% confluence before starting the osteoblast differentiation process. With a specific differentiation media [20], osteoblast differentiation was triggered after confluence attainment (Day 0). This media was made up of a culture medium enhanced with β-glycerophosphate (10 mM) and L-ascorbic acid (50 μg/mL). After that, the cells were incubated for a further 6 to 24 days. During this time, constant changes to the media were employed every two days.

To assess ALP, cells were incubated with DM media with varied amounts of CK (2.5–10 µM), after the osteoblasts differentiation and cultivated for 14 days. Following osteoblast differentiation, the cell was rinsed with PBS lysed in 0.1% Triton X-100/PBS, and the supernatants were collected by centrifugation at 12,000 rpm for 5 min. The ALP activity in the supernatants was then determined using an ALP assay kit (Sigma Chemical, St. Louis, MO, USA). To normalize the ALP activity, a BCA protein assay kit (Sigma Chemical, St. Louis, MO, USA) was used to determine the total protein concentration.

### 2.9. Calcium Deposition and ARS (Alizarin Red S) Staining

We performed the Alizarin Red S staining experiment following previously published research. In short, the cell cultures were completely rinsed with PBS on the 24th day of osteoblast differentiation, and then they were fixed with 70% ethanol. To observe and evaluate calcium accumulation, cells were stained with Alizarin Red S (ARS, 40 mM, Sigma-Aldrich) for 60 min. After the staining process, the samples were examined by applying an optical microscope (Eclipse ME600L; Nikon Instruments, Melville, NJ, USA), and representative micrographs were taken. The cells were then treated in a resolving solution (20% methanol and 10% acetic acid) for 15–18 min after drying to measure the ARS staining. The resultant solution was spectrophotometrically analyzed using an ELISA reader (TECAN, Männedorf, Zürich, Switzerland), which measured absorbance at 570 nm.

### 2.10. Collagen Content

The identical procedures utilized for the ALP assays were also employed to cultivate MC3T3-E1 cells to quantify the quantities of cellular collagen. Cells were supplemented with DM (with or without CK) to initiate the differentiation process for an additional 12 days following a 24 h incubation period and confluence. The cultural media was updated every two days.

Collagen levels were estimated using a colorimetric Sirius Red staining analysis after 12 days of treatment. Initially, the cells were washed two times with PBS and then incubated with Bouin’s solution (BS, as fixative) for 60 min. Afterward, the BS was removed, and the cells were washed with tap water for 10–15 min. The cells were left to air dry before being stained for 1 h with Sirius Red staining dye while gently shaking. Following the staining, the cells were rinsed with HCl (0.01 N) to eliminate any excess dye. The dye was then dissolved in NaOH (0.1 N), and the absorbance was measured at 550 nm using a microplate reader.

### 2.11. RNA Isolation and Real-Time Reverse Transcription-PCR (qRT-PCR) Analysis

After 3T3-L1 pre-adipocytes and MC3T3-E1 pre-osteoblasts differentiations with or without CK extract, total RNA was extracted using TRIzol reagent (Invitrogen, Waltham, MA, USA) following the instruction provided by the manufacturer. RNA content and purity were measured using spectroscopy at 260 nm and using the A260/A280 ratio. cDNA (Complementary DNA) was then synthesized from mRNA using a first strand Primescript II cDNA synthesis kit (in a 20 μL reaction using a calculated amount of RNA and the RevertAid First Strand cDNA Synthesis Kit (Takara Bio Inc., Shiga, Japan). QuantStudio 3 instrument (Applied Biosystems, Waltham, MA, USA) was used to carry out qRT-PCR with the primers provided in Supplementary Table S1. The reaction was conducted at 95 °C for 10 min, followed by 40 cycles of 95 °C for 20 s, 60 °C for 40 s, 72 °C for 40 s, and an additional expansion at 72 °C for 10 min. The target mRNA expression levels have been modified according to the household gene, β-actin.

### 2.12. Statistical Analysis

Data were presented as mean ± standard error (SE) from a minimum of three experiments. The data were analyzed with GraphPad Prism (GraphPad Software, version 8.0.2, La Jolla, CA, USA). The overall comparison between the treated and untreated (control) groups was made using statistical methods including Student’s *t*-test and two-way analysis of variance. The statistical significance level was * *p* < 0.05.

## 3. Results

### 3.1. Effect of CK on Cell Viability of 3T3-L1 and MC3T3-E1 Cells

**Figure 1:**
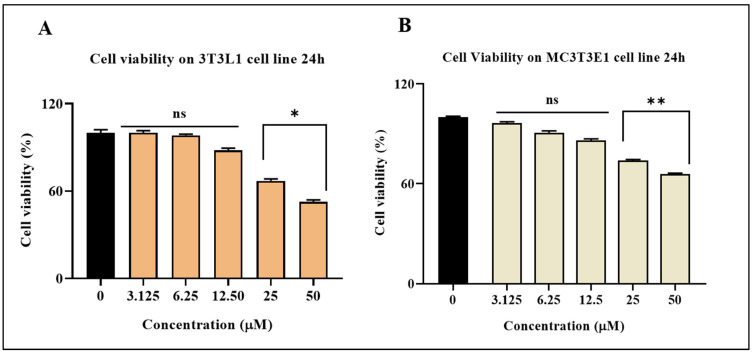
The MTT assay was used to measure cell viability. CK (3.125–50 μM) was administered to (**A**) 3T3-L1 pre-adipocyte (Orange-colored columns) and (**B**) MC3T3-E1 pre-osteoblast cells (1 × 10^4^ cells/well) (Tan-colored columns) for 24 h. A two-tailed Student’s *t*-test was used to determine whether there was a substantial distinction between the groups; ns denotes a non-significant change, * *p* < 0.05, ** *p* < 0.01 when compared to a control group (Black-colored column) that was not treated.

In the initial investigation, different doses of CK were cultivated in 3T3-L1 and MC3T3-E1 cells for 24 h to assess its effects on the growth and differentiation of these cells. After reaching >80% viability, Cells were treated with CK at 3.125, 6.25, 12.5, 25, and 50 μM of concentrations (Figure 1). The findings demonstrated that after 24 h, the greater dose of CK changed the usual structure of the cells, with some floated cells showing a harmful effect. The doses were found to have no discernible effect on the cell morphology after numerous concentration adjustments. Consequently, we chose a range of treatment doses of 2.5, 5, and 10 μM for our subsequent studies.

### 3.2. CK Prevents Lipid Formation and Triglycerides in 3T3-L1 Cells

**Figure 2:**
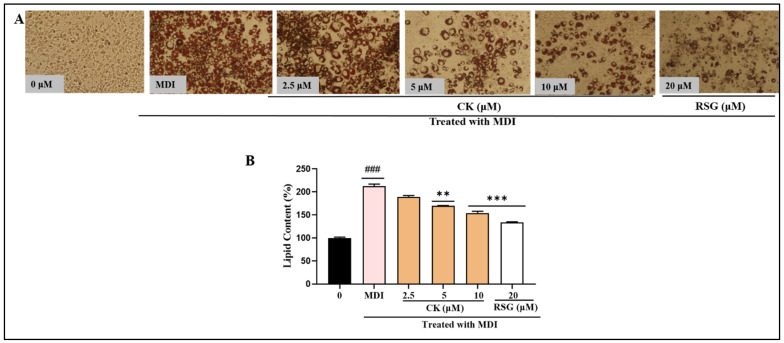
The inhibitory effect of CK on lipid accumulation in MDI-induced 3T3-L1 adipocytes was assessed as follows: (**A**) Oil Red O staining was performed to visualize fat droplets, which were then observed under a light microscope at 20× magnification. (**B**) Lipid accumulation was quantified by measuring the absorbance of Oil Red O dissolved in isopropyl alcohol at 520 nm. Data represent the mean ± SEM from three independent experiments. Statistical significance is indicated as ### *p* < 0.05, ** *p* < 0.01, *** *p* < 0.001, compared to the MDI-treated group.

Preadipocytes were converted into fully developed adipocytes by generating lipid droplets in an MDI medium. Microscopic examination showed that the CK therapy significantly decreased the intracellular formation of lipids in contrast to the MDI-treated group. The quantitative analysis showed that 100% of the cells were confluent when untreated. Furthermore, the group treated with MDI had a lipid content of almost 212.10%. In contrast to the 3T3-L1 cells treated with CK, intracellular lipid accumulation dropped by 89.42%, 69.95%, and 54.17%, respectively, when CK at doses of 2.5, 5, and 100 μM was applied. Additionally, RSG has preventive implications against obesity [21], and served as a standard drug in our study. RSG has also decreased the lipid droplets up to 34.3% (Figure 2).

### 3.3. CK Inhibits α-Glucosidase Enzyme in 3T3-L1 Cells

**Figure 3:**
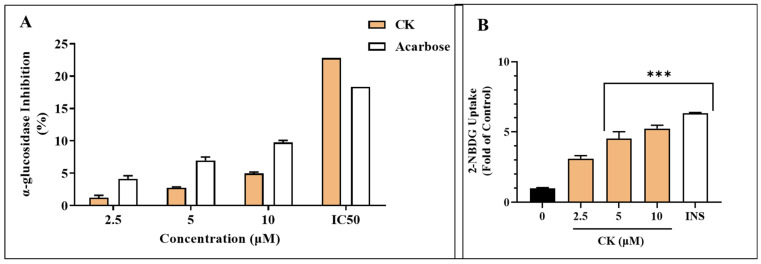
(**A**) The proportion of a-glucosidase inhibition at various CK concentrations. Acarbose, or ACR, was employed as a control. We also analyzed the half-maximum inhibitory concentration (IC_50_) of CK and ACR (**B**) 3T3L1 cells using the 2-NBDG uptake test. Using a fluorescent derivative of glucose 2-NBDG, the impact of CK on 3T3L1 cells’ glucose absorption was examined for 24 h with and without CK. Insulin was employed as a positive control at 100 nM. Data are presented as a control percentage. *** *p* < 0.001.

Obesity can be averted by inhibiting enzymes interfering with dietary fat and carbohydrate absorption in the intestine. α-glucosidase enzymes play a crucial role in converting starch into sugar. Inhibition of this enzyme inhibits the digestion of dietary fats and carbohydrates, resulting in lower calorie intake [22]. Our data depicted the ability of the CK extract to prevent the glucosidase enzyme, which was assessed. α-GLU was inhibited by the extract (Figure 3A). There was a dose-dependent response. The extract exhibited a similar profile to acarbose for α-GLU inhibition.

In the enzymatic experiment, the CK extract IC_50_ values were significantly different to the reference inhibitors. The IC_50_ for CK and acarbose, which were 22.83 and 18.34 μg/mL for the inhibition of α-GLU, respectively, were the closest to the reference inhibitor.

### 3.4. CK Stimulates 2-NBDG Uptake in 3T3-L1 Cells

Adipocytes absorb glucose when insulin interacts with insulin receptors inside the cell, which causes GLUT 4 to move to the cell surface [22]. In tests demonstrating the activation of 2-NBDG absorption into adipocytes, we validated the insulin-like and insulin-sensitizing characteristics of CK. Insulin-free CK was found to significantly improve glucose uptake compared to the control (Figure 3B). Parallel studies also examined the impact of CK on 2-NBDG absorption into adipocytes when insulin (100 nM) was present. When compared to CK without insulin, CK with insulin showed a dose-dependent substantial increase in glucose absorption.

### 3.5. Effect of CK on the Adipogenic Regulatory Factor Expression in 3T3-L1 Cells

The impact of CK on the level of expression of important regulatory genes for adipogenesis was investigated using qRT-PCR assays. In MDI-induced mature adipocytes, there was an up-regulation of adipogenic genes, such as *PPARγ*, *C/EBPα*, *LPL*, lipogenic factor *PPARγ*, *C/EBPα*, *LPL*, and a down-regulation of thermogenic gene *UCP1*. After treatment with CK, there was a potential decrease in the expressions of *PPARγ*, *C/EBPα*, *LPL*, and *FAS*, and an increase in the level of *UCP1.* The expression of *TP53* was also reduced compared to the MDI-treated group. The regulation of the expression levels of adipogenic, lipogenic, and thermogenic markers by CK was nearly distinct from the effect of RSG.

**Figure 4:**
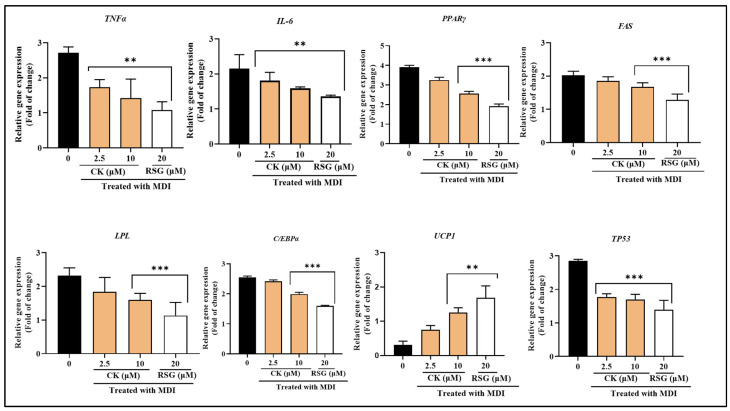
The relative expression of inflammatory genes (*TNFα*, *IL-6*), adipogenic genes (*PPARγ*, *C/EBPα*, *LPL*), the lipogenic gene *FAS*, and the thermogenic gene *UCP1*, along with *TP*^53^, was measured in differentiated 3T3-L1 cells treated with CK at concentrations of 2.5 μM and 10 μM, or RSG at 20 μM. Results are presented as the mean ± standard deviation from three independent experiments. Statistical significance was assessed using a two-tailed Student’s *t*-test. Significant differences in gene expression between untreated and treated groups are indicated as ** *p* < 0.01, and *** *p* < 0.001.

Furthermore, the growth of adipose tissue in obese situations causes a reduction in anti-inflammatory adipokines and a rise in proinflammatory adipokines, which results in issues and both local and systemic inflammation with the homeostasis of glucose [23]. Therefore, we examined whether CK can lower the mRNA level of inflammatory cytokines such as *TNFα* and *IL-6* to ascertain its impact on obesity-mediated inflammation. Our findings showed that CK treatment of differentiated adipocytes considerably decreased these cytokine levels compared to groups treated with MDI (Figure 4).

### 3.6. CK Stimulates ALP Production in MC3T3-E1 Cells

**Figure 5:**
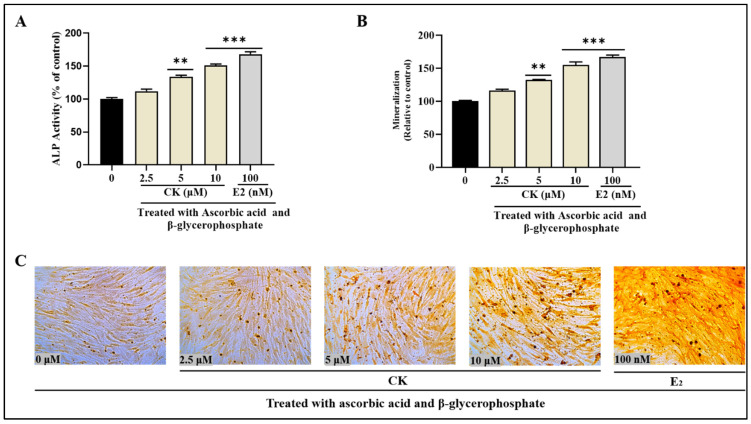
(**A**) ALP activity in CK and E_2_-treated differentiated MC3T3-E1 cells. (**B**) The impact of CK on MC3T3-E1 cell mineralization. A 96-well plate was used to measure the quantity of Alizarin Red S at 562 nm. (**C**) The calcium-binding Alizarin Red S dye was used to evaluate the calcium deposits in the extracellular matrix for matrix mineralization. Up to Day 28, CK treatment accelerated the mineralization of the extracellular matrix. The images are representative of over three different concentrations of CK and E_2._ The results are presented as the mean ± standard deviation from three independent experiments. Statistical significance was determined using a two-tailed Student’s *t*-test. Significant differences in lipid production compared to the untreated (control) group are denoted by ** *p* < 0.01, and *** *p* < 0.001.

We used a commercial instrument and according to the manufacturer’s guideline to assess the effect of CK on ALP activity. The results showed that a 14-day CK incubation significantly increased activity. Compared to control (untreated) cells, CK treatments increased ALP activity dose-dependently. Ck increased the ALP capacity by up to 150.88 ± 2.3% at 10 μM. Conversely, the standard drug E2 raised ALP activity to 168.1 ± 3.6% (Figure 5A).

### 3.7. CK Promotes Mineralization and Calcium Disposition

Extracellular matrix (ECM) mineralization plays an essential part in the development of bone because it is strongly related to osteoblast growth and differentiation [24]. To identify the effect of CK on osteoblast formation, we used ARS staining to measure the production of calcium in the extracellular matrix. In comparison to the control group, our research showed that after 14 days, CK treatment significantly increased the amount of plaque-calcified extracellular matrix. The nodules of mineralized calcium accumulation had a bright orange-red hue when stained with ARS. With CK treatment dosages that varied between 2.5 and 10 μM, the mineralization process specifically demonstrated a notable improvement, varying from 116.3 ± 2.03 to 154.6 ± 0.5%. In addition, E_2_ was used as a positive control in this research because of its proven ability to mitigate osteoporosis. The process underlying its positive benefits entails increasing osteoblast differentiation, safeguarding osteoblasts, and suppressing osteoclast activity. E2 boosted the accumulation of calcium by up to 166.9 ± 2.9% (Figure 5B,C).

### 3.8. Effect of CK on Collagen Content

**Figure 6:**
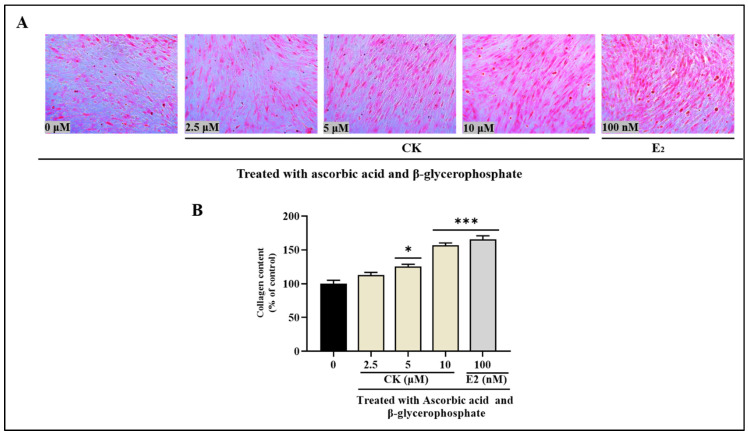
Collagen content in MC3T3-E1 cells is increased by CK extracts. For 12 days, cells were exposed to extracts at doses ranging from 2.5 to 10 μM, either with or without a differentiation medium. E_2_ was used at a concentration of 100 nm. (**A**) Picro-Sirius red staining was carried out and seen using a microscope (magnification of ×100). (**B**) Absorbance was measured at 550 nm to determine the amount of collagen. The presented data are the mean ± standard deviation (SD) of three studies. Statistical analysis revealed significant differences, denoted as * *p* < 0.1, *** *p* < 0.001 when compared with the indicated ascorbic acid and β-glycerophosphate treated group.

Sirius Red staining was used to determine collagen secretion after a two-week osteogenic development phase. Surprisingly, the data showed the beneficial effect of CK on collagen production, as seen in Figure 6. The staining levels of MC3T3-E1 cells demonstrate a distinct dose-dependent pattern. On Day 14, concentrations ranging from 2.5 to 10 μM showed significant increases of 12.9 ± 3.8%, 30.3 ± 3.3%, and 56.9 ± 3.2%. Moreover, E2 (as standard) enhanced the collagen content by approximately 65.5 ± 5.4%. These findings underscore the beneficial effects of CK therapy on collagen formation and indicate a dose-dependent amplification of the observed effects.

### 3.9. CK Regulated the Expression of Genes Involved in Osteoblast Differentiation

**Figure 7:**
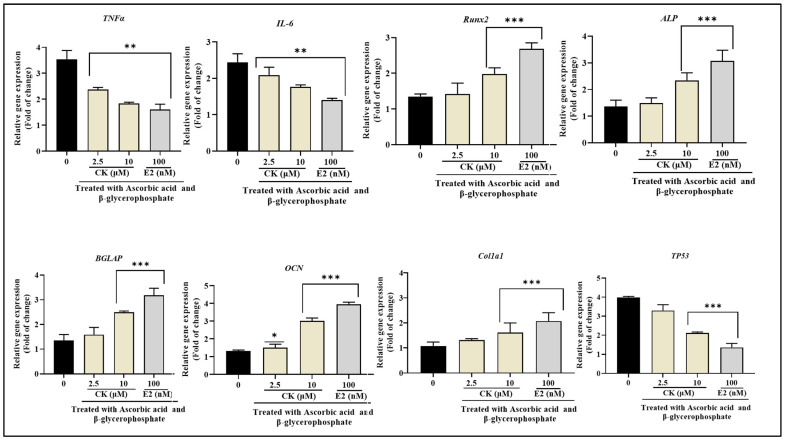
Relative expression of proinflammatory cytokines TNFα, and IL-6, and osteogenic genes *RunX2*, *ALP*, *BGLAP*, *OCN*, and *COL1a1* with *TP*^53^ in differentiated MC3T3-E1 cells on treatment with CK at 2.5 μM and E_2_ at 100 nm concentrations, respectively. A two-tailed Student’s *t*-test was used to determine whether the difference was statistically significant. The non-treated and treated groups’ significant differences in gene expression are indicated by * *p* < 0.1, ** *p* < 0.01, *** *p* < 0.001 vs. control.

We applied qRT-PCR to explore the impacts of CK on the mRNA levels of expression of osteoblast-associated targets. Figure 7 shows that CK significantly up-regulates the mRNA expression levels of cytokines associated with inflammation such as TNFα, and IL-6, and osteoblastic markers *Runx2*, *ALP*, *BGLAP*, *OCN*, and *Col1a1*, and down-regulates the main lipid and atherosclerosis pathways-related target *TP*^53^. Furthermore, previous research has shown that E_2_ decreases osteoporosis by promoting the growth and osteogenic development of MC3T3-E1 cells [19]. As a result, we used E_2_ as a positive control in our investigation. Furthermore, E_2_ significantly increased the transcription levels of osteogenic transcription factors and decreased the gene expression of *TP*^53^ in contrast to the control group.

## 4. Discussion

The growing number of age-related metabolic diseases poses a serious threat to older individuals and their quality of life. Moreover, the rising prevalence of type 2 diabetes, obesity, and osteoporosis in younger people emphasizes how urgent it is to address these global health issues [25]. According to recent research, obese people are more susceptible than non-obese people to develop osteoporosis and bone fractures [26]. The intricate relationships between obesity and bone metabolism make it imperative to comprehend the underlying mechanisms. The focus is now turning to sources of natural origin and their bioactive substances in the search for efficient treatments that control obesity and osteoporosis with minimal side effects [1]. Recently, we have reported a hypothesis of gut microbial metabolites against obesity and osteoporosis via using network pharmacology and bioinformatics approaches [15]. In that study, we performed Protein–protein interactions (PPI), gene ontology, and KEGG pathway analysis to identify target networks. The PPI indicates a strong correlation between obesity and osteoporosis with the *TP*^53^ gene. Additionally, the KEGG suggested the lipid and atherosclerosis pathway that highly linked obesity to osteoporosis. On the other side, that study suggested CK as a potential therapeutic agent to regulate obesogenic and osteoporotic transcription factors. Subsequently, the molecular docking studies demonstrated considerable negative energy (−8.0 kcal/mol) between CK and the *TP*^53^ target for both diseases, out of all metabolites. By using a 250 ns MD simulation, the conformance of CK to the intended protein *TP*^53^ was finally determined. Additionally, this study investigates several mechanisms, one of which is that excessive adipocyte development induces chronic inflammation and upsets the balance of bone remodeling, impairing bone homeostasis and causing bone loss. Thus, it was demonstrated that CK effectively reduced fat accumulation and inflammation in 3T3-L1 cells while promoting osteogenic differentiation in MC3T3-E1 cells, leading to a significant reduction in both obesity and osteoporosis.

The intricate process of adipocyte formation involves the interaction of several transcription factors and hormones. *PPARγ* and *C/EBPα* are the two main adipogenesis factors that cause adipocyte development and TG production [27]. According to experimental findings, CK inhibits the development of adipocytes in 3T3-L1 cells. In particular, 10 μM of CK has shown notable effectiveness in lowering the expression of important adipogenic markers and the buildup of intracellular lipids. Our findings demonstrated that CK efficiently increased the expression of the thermogenic gene *UCP1* and decreased adipogenic transcription factors such as *PPARγ*, *FAS*, *C/EBPα*, and the lipogenic gene *LPL*.

Adipose tissue in obesity secretes proinflammatory cytokines like *TNFα* and *IL-6*. These cytokines have been linked to osteoporosis and chronic inflammation [28]. It has long been known that *TNFα* plays a crucial role in bone homeostasis by encouraging osteoclastogenesis and inhibiting osteoblast activity. Moreover, by up-regulating *RANKL* expression in osteoblasts, fibroblasts, and T cells, *IL-6* can directly cause bone loss and promote bone resorption. Our findings showed that CK therapy dramatically decreased *TNFα* and *IL-6* mRNA levels. The imbalance between bone adipocytes and osteoblasts is another important factor in osteoporosis. There are two main stages in the process of osteoblast differentiation. ALP, a critical enzyme and phenotypic marker, is essential during the first phase. Certain osteoblast differentiation genes, including *ColA1* and *OPN*, are produced and up-regulated by ALP. Calcium deposition causes the extracellular matrix to gradually mineralize as the process moves toward its final stage [29]. Our research showed that CK therapy promotes the early development of osteoblasts, as indicated by a marked rise in ALP activity. Moreover, the process of bone mineralization is necessary to give bones their strength and hardness. Remarkably, when compared to the untreated cells, CK also significantly increases the quantity of mineralization/calcium bone structures that form in MC3T3-E1 cells. Furthermore, we assessed that CK therapy also increased the level of mRNA expression of osteogenic genes, such *as Runx2*, *ALP*, *BGLAP*, *OCN*, and *Col1a1*.

In this investigation, qRT-PCR revealed that differentiated 3T3L1 and MC3T3-E1 cells were enriched *TP*^53^. Furthermore, CK treatment demonstrated that TP53 suppression inhibited adipocyte and enhanced osteoblast differentiation in vitro, suggesting that p53 aided in the pathological advancement of obesogenesis and osteoporosis. According to these results, CK may increase osteoblastogenesis and boost bone density. Finally, this study suggested that numerous signaling pathways, including the lipid and atherosclerosis pathways, are closely related to and control the development of adipocytes and osteoblasts.

## 5. Conclusions

This study provides groundbreaking evidence that CK suppresses TP53 expression and regulates the lipid and atherosclerosis pathways to inhibit adipocytes and promote bone formation. Our results are consistent with earlier studies showing that CK might be beneficial in treating obesity and osteoporosis using network pharmacology, molecular docking, and dynamic simulation analysis. According to extensive in vitro research, CK shows promise as a cutting-edge treatment and prevention option for obesity and osteoporosis, especially for enhancing bone health in older adults. This study lays the groundwork for more research and development in this area.

## Data Availability

The data presented in this study are available on request from the corresponding authors.

## Supplementary Materials

The following supporting information can be downloaded at: https://www.mdpi.com/article/10.3390/life15010041/s1, Supplementary Table S1: Primer list used in this study.
