# Supplementary material for: Experimental Validation of Antiobesogenic and Osteoprotective Efficacy of Ginsenoside CK via Targeting Lipid and Atherosclerosis Pathways
Source: Life (Basel). 2024 Dec 31;15(1):41. doi: 10.3390/life15010041 (PMC11767077; doi:10.3390/life15010041)
Supplement: Supplementary file 1 [file life-15-00041-s001.zip › life-3380980-supplementary.pdf]

Supplementary Table S1

Primer list used in this study.

Genes	Forward Primers	Reverse Primers	Reference
PPARγ	ATGGGTGAAACTCTGGGAGATT	AGCTTCAATCGGATGGTTCTT	[1]
C/EBPα	AGGTGCTGGAGTTGACCAGT	CAGCCTAGAGATCCAGCGAC	
TNF-α	AGGGGAAATGAGAGACGCAA	TTCCCCATCTCTTGCCACAT	
IL-6	TTGACAAACAAATTCGGTACA	GAGGTGCCCATGCTACA	
FAS	TTGCCCGAGTCAGAGAAC	CGTCCACAATAGCTTCATAGC	
β-actin	CCACACCTTCTACAATGAGC	CTCGTAGATGGGCA CAGTGT	
UCP1	TAAAAACAGAAGGGCGGATG	GTGGGTTGCCCAATGAATAC	[2]
LPL	AGGACCCCTGAAGACAC	GGCACCCAACTCTCATA	[3]
TP53	TAAAGGATGCCCATGCTACAG	GACCGGGAGGATTGTGTCTC	[4]
ALP	CGAGCAGGAACAGAAGTTTGC	TGGCCAAAAGGCAGTGAATAG	[1]
Runx2	ATGGCCGGGAATGATGAGAA	TCTGTCTGTGCCTTCTTGGT	
Col-I	GATGGATTCCAGTTCGAGTATG	GTTTGGGTTGCTTGTCTG TTTG	
BGLAP	GTGCAGACCTAGCAGACACCA	GTAGCGCCGGAGTCTATTCA	[5]
OCN	GTG AGC TTA ACC CTG CTT GTG A	ACT GAA CTT GAC CGT ACA TGC GTTTGT AGG CGG TCT TC	[6]

References

1. Akter, R., et al., *In Silico and in vitro evaluation of antiobesogenic and osteoprotective effect of pomegranate juice fermented by tannin acyl hydrolase and Lactobacillus vespulae DCY75 via the Wnt/β-Catenin pathway.* ACS Food Science & Technology, 2023. **3**(11): p. 1975-1987.
2. Awais, M., et al., *Discrimination of Dendropanax morbifera via HPLC fingerprinting and SNP analysis and its impact on obesity by modulating adipogenesis-and thermogenesis-related genes.* Frontiers in Nutrition, 2023. **10**: p. 1168095.
3. Imamura, H., et al., *Resveratrol attenuates triglyceride accumulation associated with upregulation of Sirt1 and lipoprotein lipase in 3T3-L1 adipocytes.* Molecular Genetics and Metabolism Reports, 2017. **12**: p. 44-50.
4. Okita, N., et al., *Inhibitory effect of p53 on mitochondrial content and function during adipogenesis.* Biochemical and Biophysical Research Communications, 2014. **446**(1): p. 91-97.
5. Akter, R., et al., *Effect of Steamed Stauntonia hexaphylla fruit on RAW 264.7 osteoclast and MC3T3-E1 osteoblast differentiation.* 2024.
6. Lee, M.K., et al., *Regulation of osteoblast differentiation by Nurr1 in MC3T3-E1 cell line and mouse calvarial osteoblasts.* Journal of cellular biochemistry, 2006. **99**(3): p. 986-994.
